# Isokinetic muscle function, dynamic balance, and injury risk in dominant and non-dominant lower extremities of adolescent taekwondo athletes

**DOI:** 10.3389/fspor.2025.1599516

**Published:** 2025-07-14

**Authors:** Mingyuan Dong, Boseoung Kim, Jiyoung Lee, Yongchul Choi, Panpan Shi, Guanmin Zhang

**Affiliations:** Department of Physical Education, Gangneung–Wonju National University, Gangneung, Republic of Korea

**Keywords:** adolescent athletes, dominant leg, non-dominant leg, isokinetic muscle function, Y balance test, sports injuries

## Abstract

**Background:**

Adolescent Taekwondo athletes are exposed to distinct musculoskeletal demands, where imbalances in muscle function and deficiencies in dynamic balance may increase their risk of injury.

**Objective:**

This study aimed to assess the effects of Taekwondo training on isokinetic muscle function, dynamic balance, and injury risk in dominant and non-dominant lower limbs of adolescent athletes.

**Methods:**

Forty adolescent Taekwondo athletes (*n* = 40; 27 males, 13 females; mean age: 16.07 years) with an average of 7.07 years of training experience participated in this study. Participants underwent isokinetic muscle function tests (60°/s and 180°/s) and the Y-Balance Test (YBT) on both dominant and non-dominant lower limbs to assess muscular strength and dynamic balance. Physical characteristics including height, weight, and body fat percentage were also recorded.

**Results:**

Significant differences in knee extensor strength were observed between dominant and non-dominant limbs at 60°/s (*p* < 0.05), whereas flexor strength did not differ significantly. At 180°/s, significant asymmetries were found in ankle dorsiflexor strength and ipsilateral balance ratios between dominant and non-dominant sides. YBT composite scores were below the 85% threshold in several athletes, indicating an elevated injury risk. Correlation analysis showed strong associations between lower limb asymmetries and injury occurrences, especially among athletes with right-side dominance.

**Conclusion:**

These findings emphasize the critical need for bilateral neuromuscular training protocols to mitigate injury risks in adolescent Taekwondo athletes, highlighting the presence of muscle imbalances and reduced dynamic balance in this population.

## 1 Introduction

Taekwondo, a traditional Korean martial art, has evolved into a globally recognized sport since its debut as a demonstration event at the 1988 Seoul Olympic Games. It was officially included as a competitive sport at the 2000 Sydney Olympics and has since gained widespread popularity due to its unique combination of spiritual cultivation, Poomsae (forms) practice, and intense sparring competitions (1, 2). The inclusion of Taekwondo in the Olympic program has raised the physical and technical demands on athletes, leading to an increased risk of injuries during training and competition (3, 4). Studies have shown that Taekwondo athletes experience a higher incidence of injuries during competitive sparring compared to Poomsae practice, primarily due to the dynamic and high-impact nature of sparring, which involves frequent physical contact and rapid, forceful movements (3, 5). Furthermore, the majority of Taekwondo practitioners report sustaining injuries during training or competition, with overuse injuries and muscle imbalances being significant contributing factors (4–7).

The technical demands of Taekwondo differ significantly from those of traditional track and field sports. Taekwondo athletes often adopt a stance that emphasizes unilateral loading, with one leg positioned forward and the other backward, leading to asymmetrical muscle development and potential imbalances (8, 9). This unilateral loading pattern is similar to that observed in other sports such as tennis, golf, and badminton, where repetitive use of one side of the body can lead to muscle imbalances and increased injury risk (10, 11). Muscle imbalances in the lower extremities can be quantified using the bilateral balance ratio (comparing strength between the left and right limbs) and the ipsilateral balance ratio (comparing the strength of agonist and antagonist muscle groups, such as the hamstrings and quadriceps). An optimal hamstring-to-quadriceps ratio (H: Q) is approximately 0.6 to 0.8, and deviations from this ratio, as well as bilateral strength differences exceeding 8%–10%, are associated with increased injury risk and reduced athletic performance (12–14). These imbalances can negatively affect agility, balance, and overall performance, which are critical for Taekwondo athletes (9, 15).

Isokinetic muscle function testing, which has been widely used since the late 1960s, provides a reliable method for assessing muscle strength and imbalances (16, 17). Additionally, the Y-balance test is a validated tool for evaluating dynamic balance, proprioception, and integrated motor function, making it particularly useful for assessing the functional capabilities of Taekwondo athletes (8, 18). Adolescence is a critical period for physical and motor development, and balanced training is essential to ensure the harmonious development of physical functions (19).

In this study, limb dominance was determined by asking participants which leg they preferred to use for kicking, which is a standard method in martial arts research (9). The dominant lower extremity (DLE) was defined as the preferred kicking leg, while the non-dominant lower extremity (NDLE) functioned primarily in support and balance. This asymmetrical training can lead to muscle imbalances, which not only impair athletic performance but also increase the risk of injuries such as ligament sprains, muscle strains, and joint pain (9, 20). Previous studies have also indicated performance differences between dominant and non-dominant limbs in terms of strength, coordination, and balance, but there is limited evidence in adolescent Taekwondo populations (21, 22). Building on this foundation, it is crucial to clarify terminology and consolidate normative benchmarks to better understand the link between asymmetrical training and injury risk. Despite the high prevalence of injuries in Taekwondo, terms like “far-leg injury” have been inconsistently used in prior literature and are avoided here for clarity. There is limited research on the relationship between muscle imbalances, motor function, and injury risk in adolescent Taekwondo athletes. Most studies have focused on the types, locations, and causes of injuries in Taekwondo competitions, with little attention given to the role of muscle imbalances and their impact on injury risk (1, 5, 6, 23). To enhance clarity, a summary table (Table 1) has been added to present normative strength ratios (e.g., optimal H:Q ratio and bilateral strength difference thresholds) and commonly referenced injury risk cut-offs for the Y-balance test (Table 1).

**Table 1:** Normative strength ratios and injury risk thresholds.

Variable	Normative range/threshold	Implication	Reference
Hamstring-to-Quadriceps Ratio (H: Q)	0.6–0.8	Lower ratios linked to increased risk of hamstring strain	(12, 13)
Bilateral Strength Difference	≤10%	Differences >10% increase risk of lower limb injury	(14)
Ankle Plantarflexion/Dorsiflexion Ratio	3:1 (Plantarflexors ∼3× stronger than dorsiflexors)	Imbalance may affect postural control and mobility	(24)
Y-Balance Test Composite Score (normalized to leg length)	≥85%	Scores <85% indicate higher injury risk	(18, 25)
Anterior Reach Asymmetry (YBT)	<4 cm	Asymmetries >4 cm associated with increased injury risk	(18)

Therefore, this study aims to evaluate the isokinetic muscle function and Y-balance performance of adolescent Taekwondo athletes, with a focus on the dominant and non-dominant lower extremities. By examining the relationship between muscle imbalances, dynamic balance, and injury risk, this study seeks to provide evidence-based recommendations for injury prevention and performance optimization in adolescent Taekwondo athletes.

## 2 Materials and methods

### 2.1 Participants

The study sample included 40 participants (*n* = 40), with 27 males and 13 females’ taekwondo athletes, from middle and high schools in Gangwon Province, South Korea. The adolescent taekwondo athletes who participated in the experiment fully understood the purpose and procedure of the study and volunteered to participate in the experiment. The characteristics of experimental subjects are shown in Table 2.

**Table 2:** Physical characteristics of the study subjects.

N	Age (yr)	Experience (yr)	Height (cm)	Weight (kg)	Body fat (%)
27	16.07 ± 0.94	7.07 ± 2.27	178.08 ± 5.64	68.94 ± 9.60	12.24 ± 4.39
13	16.07 ± 0.984	7.07 ± 1.76	167.66 ± 4.85	63.50 ± 9.35	26.60 ± 5.18

Dominance was identified through a self-report question asking which leg the athlete prefers to use when executing a Taekwondo kick (e.g., roundhouse or back kick), which is a standard criterion in martial arts research (9).

### 2.2 Measuring items and tools

#### 2.2.1 Measuring physique and body composition

A bioresistance analyzer (Inbody 3.0, Biospace, Korea) was used to measure the subjects’ height, weight, and body fat (%) during fasting for more than 10 h. Inbody, a method of measuring bioelectrical impedance in different parts of the body, requires the subject to stand barefoot on a metal plate equipped with sensors and gently press the electrode sensor on the electrode handle with both hands, keeping the underarms of both hands apart.

#### 2.2.2 Isokinetic muscle function test

To minimize fatigue and test order effects, all subjects followed a standardized test sequence, starting with isokinetic strength tests followed by balance testing. The dominant limb was tested before the non-dominant limb for all participants, and a sufficient rest interval (2–3 min between repetitions, and 3 min between limbs) was ensured throughout.

Before starting the test, the subjects warmed up on the dynamometer for 10 min. This reduced warm-up duration was chosen to maintain test feasibility while still preparing the neuromuscular system. After a 2 min break, the protocol was executed. Isokinetic muscle function tests in the knee and ankle joints were measured using an isokinetic dynamometer (Humac Norm, CSMi, Stoughton, MA, USA). The test was set to uniaxial muscle contraction and measured at 60°/s(Nm/kg) and 180°/s(watts/kg). The angle of the chair is adjusted to 100°, and the motion axis of the knee joint is parallel to the motor side of the device. For each test, the range of motion of the lower limbs was first measured and determined prior to initiating the isokinetic assessment. To prevent accidents, the test chair is fitted with safety pins and secured with a belt to prevent movement of the body.

First, participants were asked to choose their preferred leg, place the knee joint at 90 degrees, and then do their best to extend and bend the knee while giving the signal. During the test, three rehearsals, four 60°/s repetitions, and four 180°/s repetitions were performed. In between each speed, participants rested for at least 2–3 min, and after completing the test on one leg, they were tested on the other leg. To calculate the Ipsilateral muscle strength ratio, the maximum flexor torque was divided by the maximum extensor torque and used for subsequent analysis (23, 26, 27).

In the ankle isokinetic test, the athlete is required to sit in a performance chair with the torso at 70°, the hip joint and knee joint bent to 90°, and the ankle joint aligned with the sole of the foot (plantar flexion) by 10–15°. The foot pedals are secured with two crossed straps, and the torso, pelvis, and thighs (distal third) are secured with straps to prevent compensatory movement. Athletes pre-practice three maximum repetitions (50% of maximum effort) at two speeds in each test to familiarize themselves with the procedure and warm up. During the test, it is specified to perform a maximum of three plantar bends and back extensions at each speed. Set a 2 min rest period between the two speed assessments and a 3 min rest period between the dominant and non-dominant ankle assessments. At the same time during the process, the adolescent taekwondo athletes are tested at maximum intensity with verbal stimulation and encouragement from the same examiner (28).

#### 2.2.3 Y-balance test

Dynamic balance tests are performed using a YBT (Y-Balance test) device (FMS TM, Chatham, VA, USA) according to recommended guidelines. After the isokinetic muscle strength test, the participants rested for 20 min. The examiner demonstrated YBT and gave instructions to the subjects. Each subject’s foot is placed on the examination table, one foot is fixed in the center, and the subject is stretched to the maximum extent in the front, posterolateral, posterolateral directions. Test the dominant leg first, then the non-dominant leg. The examiner provides verbal instructions and signals to begin. Each side was tested 3 times and the maximum value was recorded. Record in 0.5 cm increments. If the tester’s feet touch the ground due to loss of balance, the test is redone after explanation. The tests were conducted indoors in quiet conditions to avoid environmental disturbances. The examiner looks over the subject’s shoulder to make sure the subject is not distracted (18, 29).

The total score was calculated by measuring the length of the lower limb from the anterior superior iliac bone to the middle of the medial ankle bone with a tape measure. The total score is calculated as follows.

{(anterolateral+posterolateral+posteromedial)÷(3×lowerlimblength)}×100

### 2.3 Term definitions

#### 2.3.1 Peak torque

Peak torque is the maximum amount of work done by the muscle, that is, the maximum amount of muscle force, using feet or nm as a unit of measurement.

#### 2.3.2 Average power

It is an analysis of the two directions of motion and is calculated by dividing the total work by the contraction time in watts.

#### 2.3.3 Peak torque to weight ratio

Refers to the amount of muscle strength that can be exerted by kg weight.

#### 2.3.4 Ipsilateral muscle strength ratio

The ratio of the strength of the extensor and flexor muscles of the lower extremity is calculated as the peak extensor torque/peak flexor torque ×100, expressed as a percentage.

#### 2.3.5 Bilateral balance ratio

The ratio of strength between the left and right sides of the body, if the difference is more than 10%, the probability of injury will increase.

### 2.4 Test flow chart

**Figure 1:**
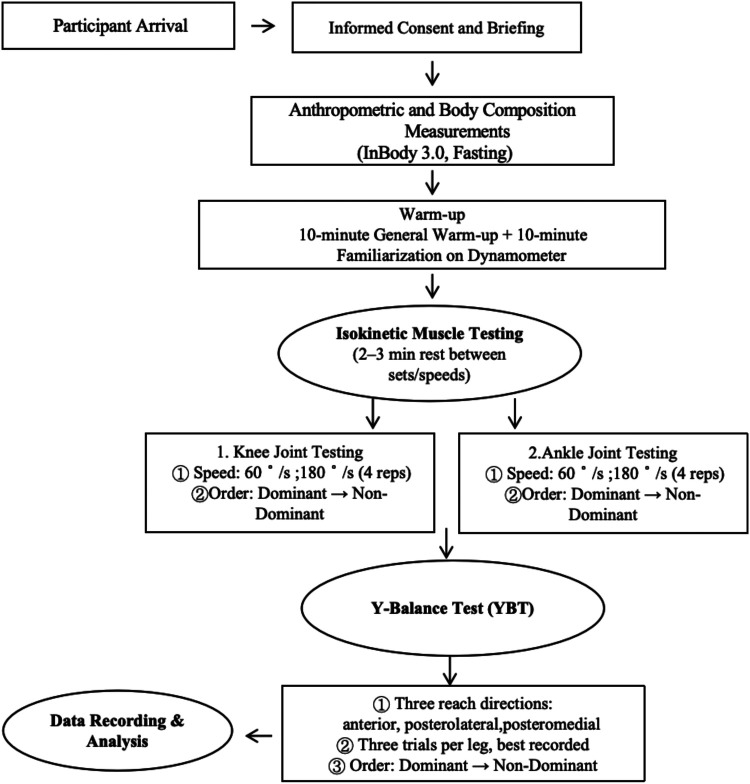
Flow chart of the experimental protocol.

To clarify test order and timing, a flow chart of the full testing protocol has been added (Figure 1), showing the sequence of warm-up, isokinetic strength testing (knee, then ankle), followed by a 20-minute rest and Y-balance testing.

### 2.5 Statistical analysis

Sample size was determined using G*Power 3.1 software (30, 31). with an effect size of 0.4(Cohen’s f), power of 0.80, and alpha of 0.05 for ANOVA repeated measures, indicating that a minimum of 36 participants was required. We recruited 40 to account for potential dropouts.

The SPSS 26.0 program was used to calculate the mean (M) and standard deviation (SD) of all data. The isokinetic muscle function and Y balance of dominant and non-dominant lower limbs of Taekwondo athletes were analyzed by independent sample *T*-test. Chi-square tests were used to analyze the correlation between dominant and non-dominant lower extremity injuries. All statistical significance levels were set at *p* < 0.05.

## 3 Results

### 3.1 Isokinetic muscle function of knee joint

The isokinetic muscle function test results of dominant and non-dominant lower extremity knee joint of adolescent taekwondo athletes are shown in Table 3. At 60°/s, the left dominant lower extremity (LDLE) group shows significantly higher muscle function values in the left extensor compared to the right dominant lower extremity (RDLE) group (*p* = 0.025). At 180°/s, the differences in muscle function values between the RDLE and LDLE groups are not statistically significant (*p* = 0.164).

**Table 3:** Comparison of isokinetic muscle function of the knee joint.

Test items	Variable	RDLE (*N* = 21)	LDLE (*N* = 19)	*P*
Isokinetic knee 60°/s, peak Nm/kg, %	Right extensor	184.8 ± 51.8	190.6 ± 39.3	0.197
	Left extensor	174.5 ± 55.6	183.8 ± 34.5	0.025*
	Left and right ratio	10.4 ± 6.2	7.9 ± 5.1	0.147
	Right flexor	83.5 ± 29.7	73.8 ± 27.3	0.642
	Left flexor	85.9 ± 29.8	86.3 ± 19.9	0.113
	Flexor left and right ratio	1.6 ± 3.8	6.7 ± 4.7	0.47
	Right extensor (% weight)	276.2 ± 56.2	268.0 ± 174.8	0.244
	Left extensor (% weight)	266.0 ± 61.0	267.6 ± 46.8	0.289
	Right flexor (% weight)	126.6 ± 38.8	102.7 ± 40.7	0.899
	Left flexor (% weight)	130.1 ± 37.5	126.3 ± 29.8	0.279
	Right flexor/extensor (%)	45.7 ± 8.9	38.0 ± 10.2	0.227
	Left flexor/extensor (%)	48.8 ± 7.4	46.9 ± 8.6	0.935
Isokinetic knee 180°/s, average Watts/kg, %	Right flexor	196.4 ± 50.9	197.0 ± 47.7	0.396
	Left extensor	187.4 ± 57.7	196.1 ± 43.5	0.053
	Right flexor	106.4 ± 37.1	95.0 ± 27.1	0.087
	Left extensor	104.0 ± 35.9	101.5 ± 27.6	0.118

Data are presented as Mean ± SD.

RDLE, right-dominant lower extremity; LDLE, left-dominant lower extremity; Nm, newton meter.

* *p* < 0.05 indicates a significant difference within the experimental group.

** *p* < 0.01 indicates a highly significant difference within the experimental group.

**Figure 2:**
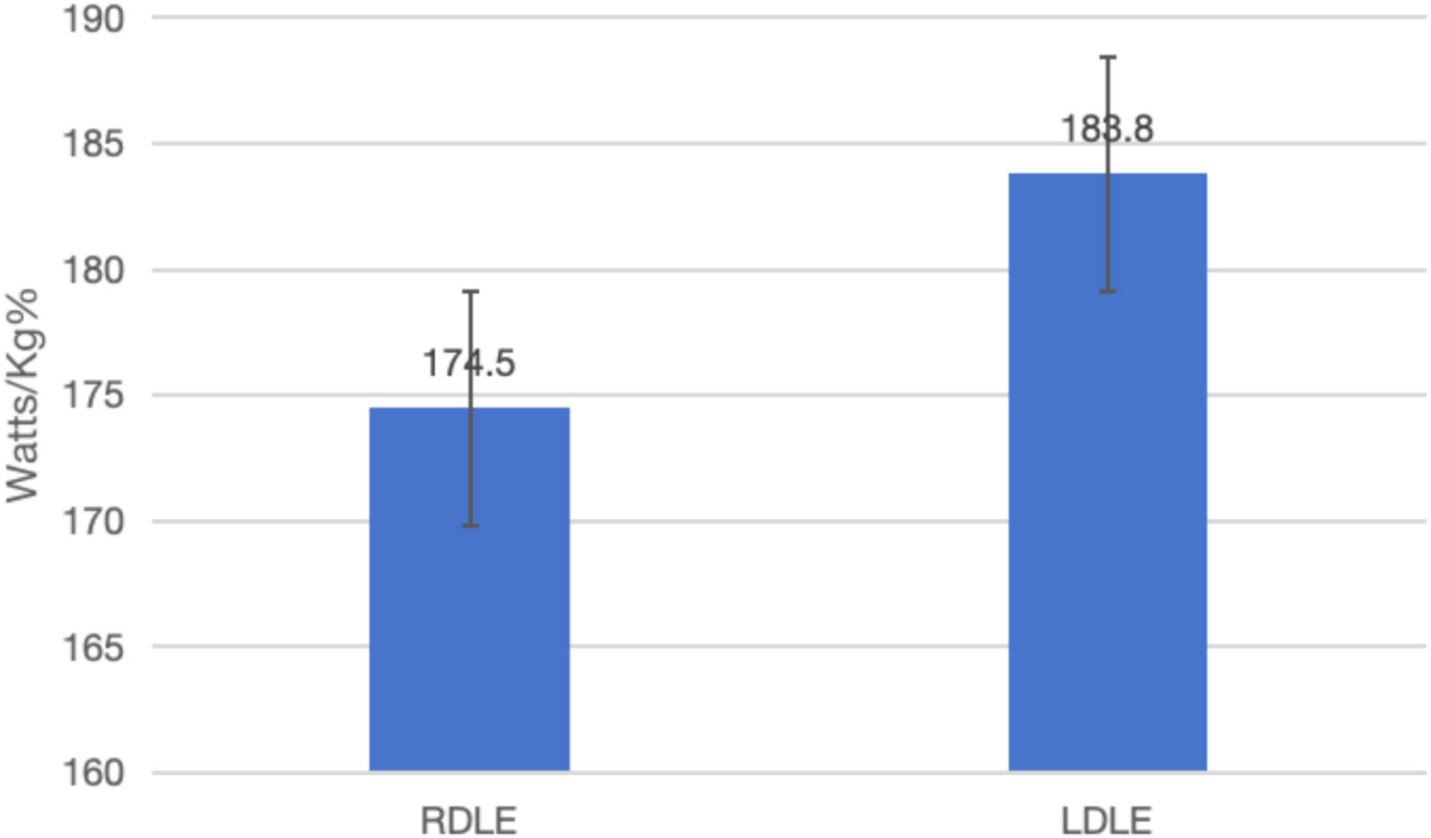
Comparison of knee joint 60°/s extensors.

Figure 2 illustrates that the peak torque of the left knee extensor in the RDLE group was 174.5 ± 55.6 Nm, while in the LDLE group it was 183.8 ± 34.5 Nm, with a significant difference (*p* = 0.025). This result indicates that the left-dominant lower extremity group demonstrated superior isokinetic extensor strength at 60°/s.

### 3.2 Isokinetic muscle function of ankle joint

The isokinetic muscle function test results of ankle joint of dominant and non-dominant lower limbs of adolescent taekwondo athletes are shown in Table 4. At 60°/s, most *p*-values were greater than 0.05, indicating no significant difference between the RDLE and LDLE groups in these muscle functions. However, at 180°/s, the *p*-values for right and left plantar flexion were both *p* < 0.01, indicating a statistically significant difference between the two groups. Specifically, the LDLE group has higher muscle function values in these aspects compared to the RDLE group.

**Table 4:** Comparison of isokinetic muscle function of the ankle joint.

Isokinetic muscle function indicator	Variable	RDLE (*N* = 21)	LDLE (*N* = 19)	*P*
Isokinetic Joint angle 60°/s, peak Nm/kg, %	Right Plantar Flexors	54.2 ± 5.3	54.8 ± 6.1	0.792
	Left Plantar Flexors	56.4 ± 6.1	59.3 ± 5.4	0.509
	Right Dorsiflexors	45.5 ± 20.1	47.7 ± 17.1	0.205
	Left Dorsiflexors	35.4 ± 13.7	34.2 ± 12.3	0.839
Isokinetic Joint angle 180°/s, average Watts/kg, %	Right Plantar Flexors	48.5 ± 6.6	48.0 ± 7.2	0.157
	Left Plantar Flexors	52.2 ± 5.9	52.5 ± 4.4	0.518
	Right Dorsiflexors	60.9 ± 20.7	72.1 ± 12.8	<0.01**
	Left Dorsiflexors	52.8 ± 22.2	56.7 ± 16.9	<0.01**

Data are presented as Mean ± SD.

RDLE, right-dominant lower extremity; LDLE, left-dominant lower extremity; Nm, newton meter.

* *p* < 0.05 indicates a significant difference within the experimental group.

** *p* < 0.01 indicates a highly significant difference within the experimental group.

**Figure 3:**
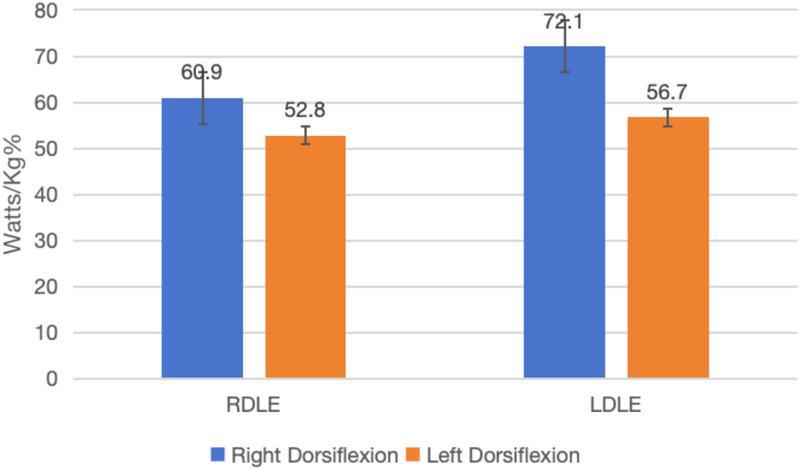
Comparison of 180°/s dorsiflexion of ankle joint.

Figure 3 visually confirms the greater plantar flexion strength of the LDLE group at 180°/s. The isokinetic muscle function of the ankle joint in adolescent taekwondo athletes shows some differences between the dominant and non—dominant lower limbs, especially at higher velocities (180°/s). The LDLE group tends to have higher muscle function in certain aspects, which may have implications for training and injury prevention strategies.

### 3.3 Y-balance test

The Y-balance test results are shown in Table 5. All *p*-values were greater than 0.05, indicating no statistically significant differences between the dominant and non-dominant lower limbs in terms of balance performance. However, the *p*-values for “Right Rear Outer” (0.096) and “Left Rear Outer” (0.059) suggest a trend toward a difference, with the left dominant lower extremity performing slightly better in the outer rear balance directions.

**Table 5:** Y-balance test comparison.

Y-balance test items	Variable	RDLE (*N* = 21)	LDLE (*N* = 19)	*P*
Y-balance	Right Front (cm)	59.2 ± 6.7	62.3 ± 5.3	0.406
	Left Front (cm)	58.0 ± 7.4	60.8 ± 6.4	0.597
	Right rear inner (cm)	95.6 ± 8.0	95.9 ± 8.7	0.609
	Left rear inner (cm)	95.5 ± 8.1	95.1 ± 7.9	0.629
	Right rear outer (cm)	96.9 ± 6.3	99.1 ± 8.3	0.096
	Left rear outer (cm)	97.1 ± 10.5	99.0 ± 7.1	0.059
	Right reception (%)	83.8 ± 5.8	84.1 ± 5.0	0.161
	Left reception (%)	83.3 ± 6.7	83.3 ± 5.3	0.078

Data are presented as Mean ± SD.

RDLE, right-dominant lower extremity; LDLE, left-dominant lower extremity; Nm, newton meter.

Overall, there are no significant differences in the Y-balance test results between the right and left dominant lower extremities in these adolescent taekwondo athletes. The data suggest that both limbs demonstrate similar balance capabilities, with some indications of potential slight differences in rear outer balance.

### 3.4 Correlation between dominant lower limb injury and non-dominant lower limb injury

The relationship between injuries in the dominant and non-dominant lower limbs is presented in Table 6. By analyzing the correlation between dominant lower limb injury and non-dominant lower limb injury of adolescent taekwondo athletes, it was found that there was a significant statistical difference between them (*p* < 0.01).In terms of dominant and non-dominant lower extremity injuries, athletes with the right dominant leg had a 24% chance of sustaining a right leg injury, while athletes with a left dominant leg had a 20% chance of sustaining a left leg injury.

**Table 6:** Injury relationship of the dominant lower extremity.

Dominant lower extremity group & sample size	Injury location	Frequency	*P*
RDLE (*N* = 21)	Right leg injury	9	<0.01**
	Left leg injury	0	
	No injury	12	
LDLE (*N* = 19)	Right leg injury	0	
	Left leg injury	8	
	No injury	11	

* *p* < 0.05 indicates a significant difference within the experimental group; *p *<* *0.01 indicates a highly significant difference within the experimental group*; *Data are presented as Mean* *±* *SD.*

RDLE, right-dominant lower extremity; LDLE, left-dominant lower extremity; Nm, newton meter.

From Table 6, in the RDLE group, the frequency of right leg injuries is higher, while the frequency of Left leg injuries is 0. In the LDLE group, the frequency of left leg injuries is higher, while the frequency of right leg injuries is 0. The *p*-value < 0.01 indicates a significant correlation between dominant lower—limb injuries and non—dominant lower—limb injuries in the RDLE group. The results show that there is a correlation between dominant lower—limb injuries and non—dominant lower—limb injuries among adolescent taekwondo athletes. Specifically, athletes in the RDLE group are more likely to have right—leg injuries, while athletes in the LDLE group are more likely to have left leg injuries.

## 4 Discussion

### 4.1 Isokinetic muscle function of the knee joint

Isokinetic muscle function assessment of the knee joint is a reliable and objective method for evaluating muscle performance, including strength, power, and endurance, and is widely used in injury prediction and rehabilitation (32, 33). In sports with strong unilateral movements, such as badminton, golf, and tennis, athletes often exhibit higher rates of muscle imbalance (10). While numerous studies have investigated the isokinetic knee muscle strength of adolescent Taekwondo athletes, focusing on bilateral and ipsilateral proportions (34–38). However, research specifically addressing lower limb asymmetry and the balance between the dominant and non-dominant legs in Taekwondo remains limited.

In this study, no significant difference was observed in isometric muscle strength between the right dominant lower extremity (RDLE) and left dominant lower extremity (LDLE) at angular velocities of 60° and 180°. However, the LDLE group exhibited greater maximum muscle strength and average extensor power compared to the RDLE group. Specifically, the extensor strength in the LDLE group was significantly higher than in the RDLE group, contrasting with findings by Harbili et al. (39), who reported no bilateral asymmetry in knee joint strength among competitive Taekwondo athletes. Conversely, Čular et al. (21) observed significant differences in muscle strength between the left and right sides in both competitive and non-competitive Taekwondo athletes. In addition, studies have shown that differences in lower limb isokinetic muscle strength are also observed in some healthy populations, and these variations have been associated with age and gender factors (40). This inconsistency could reflect differences in athlete age, experience, or training emphasis. For example, adolescent athletes may experience greater asymmetry due to neuromuscular immaturity and incomplete physical development (41). It is important to note that while a speculative interpretation suggests the smaller number of LDLE athletes might result from long-term training against RDLE opponents, this remains hypothetical and should be tested in future studies. The study revealed that the left-right ratio of extensor muscle strength was 10.4% in the RDLE group and 7.9% in the LDLE group, while the bilateral leg differences in flexor muscle strength in the LDLE group was 6.7%. These findings suggest a higher potential for injury in both dominant and non-dominant lower limbs, as muscle strength differences exceeding 10% are associated with increased injury rates (42–44). The significant differences in extensor strength between the two groups and in flexor strength within the LDLE group highlight the need for intervention strategies to improve bilateral muscle balance in adolescent Taekwondo athletes.

Although no difference was found in the ipsilateral muscle strength ratio between the RDLE and LDLE groups, the ratio in both groups was significantly below 60%. The LDLE group exhibited a notable difference between the dominant and non-dominant sides. While well-trained athletes typically show minimal differences in ipsilateral muscle strength ratios (45), these findings align with observations in teenage male soccer players at lower training levels (32, 46). The observed disparities in lower extremity strength ratios may stem from musculoskeletal immaturity and underdeveloped neuromuscular control in adolescent Taekwondo athletes.

### 4.2 Isokinetic muscle function of the ankle joint

Taekwondo athletes are constantly jumping, stepping, attacking, and defending; therefore, the ankle joint is frequently subjected to external forces. Strengthening ankle joint muscles is essential for maintaining stability and reducing injury risk. When the torque ratio of ankle dorsiflexion to plantarflexion is about 30%–40%, the muscle strength balance between agonists and antagonists is considered optimal (47). Therefore, enhancing dorsiflexor strength plays a crucial role in injury prevention.

Under the condition of 60°/s angular velocity, no significant differences in ankle muscle strength were found between dominant and non-dominant limbs. However, at 180°/s angular velocity, the LDLE group had significantly higher dorsiflexion difference on the dominant ankle than the RDLE group. The mechanical implications of such dorsiflexor dominance suggest reduced joint stiffness during landing, potentially increasing ankle sprain susceptibility (48). The difference in the maximum strength ratio of the plantar flexors and dorsiflexors of the dominant and non-dominant ankles may be associated with an increased incidence of injury to the right ankle, as the maximum strength ratio of the left ankle is 30%–40%, while that of the right ankle is less than 20%.

The maximum torque ratio between plantarflexors and dorsiflexors was less than 20% in the right ankle but fell within the optimal 30%–40% range in the left ankle. This imbalance may indicate a greater susceptibility to right ankle injuries. This interpretation is consistent with previous findings in volleyball players, where the non-dominant ankle showed lower strength at 60°/s angular velocity (28). Although these findings suggest potential injury risk, they should be interpreted with caution due to the lack of direct injury data. Further research with biomechanical and longitudinal injury tracking is recommended.

### 4.3 Y balance test

The Y Balance Test (YBT) is a widely utilized tool for assessing proprioception, stability, balance, and symmetrical balance of the body. A reach difference greater than 4 cm between sides is associated with a 2.5-fold increase in lower limb injury risk (49), while composite scores below 89% indicate reduced dynamic stability. While some studies have reported that male athletes tend to achieve higher composite YBT scores compared to female athletes (50), there is limited research on differences in YBT performance among athletes from various sports disciplines. This gap highlights the need for sport-specific analyses to better understand the relationship between balance performance and injury risk.

In this study, no significant differences were observed between RDLE and LDLE groups. However, the composite scores for both were below 85%, and side-to-side differences were below 4 cm. While the reach difference may not independently indicate risk, the consistently low composite scores are concerning. These results may suggest deficits in neuromuscular control and postural stability, particularly since adolescent athletes are still developing proprioceptive function (51, 52). Importantly, although no statistical difference was found between groups, the absolute YBT performance level was poor, indicating a potentially high injury risk. This underscores the need for incorporating targeted dynamic balance and neuromuscular training in Taekwondo development programs (53).

In conclusion, the YBT results from this study indicate a potential increased risk of lower limb injury among the participants, as evidenced by composite scores below 85% and side-to-side reach differences within the critical threshold. These findings underscore the need for targeted balance training and injury prevention strategies, particularly in populations with similar performance profiles. Speculation on causality—for instance, linking low YBT performance directly to training asymmetry—should be framed as hypothesis-generating rather than conclusive. Longitudinal studies would help establish the predictive value of YBT scores more clearly. Future research should explore sport-specific differences in YBT performance to further refine injury risk assessment and prevention protocols.

### 4.4 Injury correlation between dominant lower limb and non-dominant lower limb

Injury analysis revealed differing patterns between the RDLE and LDLE groups. The RDLE group had more right-side injuries, while the LDLE group had more left-side injuries, suggesting limb dominance may influence injury site.

These findings are consistent with previous studies. For example, Haddad et al. (54) showed that limb dominance impacts knee and ankle injury distribution. Neuromuscular asymmetry and load distribution may be key contributing factors, particularly in sports requiring unilateral skills.

However, this conclusion remains correlational. The cross-sectional nature of this study limits causal inference, and future prospective studies are needed. Additionally, Zhao et al. (55) emphasized that asymmetrical strength and coordination are injury risk factors, supporting our recommendation to improve bilateral symmetry. Targeted training such as unilateral strength and balance exercises may improve neuromuscular control (56), especially in adolescent athletes with clear limb dominance. This could reduce future injury incidence by enhancing motor control and joint stabilization.

In conclusion, the correlation between limb dominance and injury patterns in adolescent Taekwondo athletes highlights the importance of evaluating bilateral and ipsilateral balance ratios. These findings provide valuable insights for developing tailored training and injury prevention programs aimed at reducing the risk of lower limb injuries in this population.

### 4.5 Limitations

This study has several limitations that should be considered when interpreting the findings. First, the cross-sectional design prevents causal inference, making it unclear whether the observed muscle imbalances and balance performance are causes or consequences of injury. Longitudinal research is needed to explore the developmental trajectory of these factors and their long-term relationship with Taekwondo training. Additionally, the retrospective injury data based on self-reports and athlete records may be subject to recall bias, affecting the reliability of injury type, frequency, and timing. Future studies employing prospective tracking and standardized diagnostic methods are recommended to enhance data accuracy.

Second, the homogeneity of the sample—limited to South Korean adolescent Taekwondo athletes—reduces the generalizability of the results to other age groups, sports, or cultural contexts. The absence of a control group also limits the ability to discern whether the observed patterns are unique to Taekwondo or reflective of general adolescent development. Including diverse athletic populations and non-athlete controls in future research would help clarify the sport-specific effects of Taekwondo training.

## 5 Conclusion

The aim of this study was to analyze the effects of isokinetic muscle function, Y balance, and injury on dominant and non-dominant lower limbs of adolescent taekwondo athletes by performing isokinetic muscle function tests and Y balance tests on knee and ankle joints. The results showed that there were differences between the dominant and non-dominant limbs in the extensor muscles of the knee joint and the dorsiflexor muscles of the ankle joint. In addition, both the right and left dominant lower limb groups showed higher muscle imbalance in terms of bilateral and ipsilateral balance ratios, and a lower-than-normal injury incidence threshold based on the Y-balance composite score. Therefore, it is necessary to implement targeted intervention programs aimed at the balanced development of lower limb muscle function to reduce the risk of injuries caused by muscular imbalance.

Based on our findings, we recommend that coaches and clinicians incorporate unilateral strength training protocols focusing on the weaker (non-dominant) limb, particularly emphasizing knee extensor and ankle dorsiflexor strengthening. Furthermore, neuromuscular training programs that integrate dynamic balance exercises, such as single-leg stance Y-balance drills and proprioceptive coordination tasks, should be regularly applied to improve symmetry and enhance joint stability. In particular, screening protocols such as routine isokinetic testing and the YBT should be systematically implemented to identify at-risk athletes early. Regular monitoring of limb asymmetry using isokinetic and YBT assessments is also advised to ensure timely adjustments in training loads and to track progress over time. Coaches are also advised to design individualized training interventions tailored to each athlete’s muscle imbalance profile—for example, enhancing hamstring strength relative to quadriceps where needed or reinforcing the non-dominant limb. To promote bilateral symmetry, exercises should be incorporated that equally challenge both limbs across different movement planes. Finally, injury prevention strategies should include structured warm-up and cool-down routines as well as progressive loading plans adapted to the specific neuromuscular demands of Taekwondo and the developmental stage of adolescent athletes.

## Data Availability

The raw data supporting the conclusions of this article will be made available by the authors, without undue reservation.
